# Physicochemical Composition and Bioactive Properties of Uruguayan Bee Pollen from Different Botanical Sources

**DOI:** 10.3390/foods14101689

**Published:** 2025-05-10

**Authors:** Adriana Gámbaro, Marcelo Miraballes, Nahir Urruzola, Maximiliano Kniazev, Cecilia Dauber, Melissa Romero, Adriana Maite Fernández-Fernández, Alejandra Medrano, Estela Santos, Ignacio Vieitez

**Affiliations:** 1 Department of Food Science and Technology, School of Chemistry, Universidad de la República (UdelaR), Montevideo 11800, Uruguay; agambaro@fq.edu.uy (A.G.); mmiraballes@fq.edu.uy (M.M.); urruzola@fq.edu.uy (N.U.); mkniazev@fq.edu.uy (M.K.); cdauber@fq.edu.uy (C.D.); melissaromero@fq.edu.uy (M.R.); afernandez@fq.edu.uy (A.M.F.-F.); amedrano@fq.edu.uy (A.M.); 2 Ethology Section, School of Sciences, Universidad de la República (UdelaR), Montevideo 11400, Uruguay; estelasantos@gmail.com

**Keywords:** bee pollen, bioactive properties, metabolic syndrome, obesity, type 2 diabetes

## Abstract

Bee pollen is widely recognized for its health benefits, with its nutritional and bioactive properties varying by botanical origin. This study analyzed twelve bee pollen samples collected from six different apiaries in Uruguay during two seasons (spring and autumn) to determine their botanical composition; nutritional profile (protein, lipids, carbohydrates, dietary fiber, ash, and fatty acid profile); bioactive compound content (total phenols, vitamin C, tocopherols, and carotenoids); antioxidant activity (ABTS and ORAC); color; and ability to inhibit enzymes involved in carbohydrate and fat digestion. Among the samples collected in autumn, three were monofloral (one from *Casuarina* and two from *Eucalyptus*). The spring samples, however, were all multifloral, except for one monofloral *Rapeseed* sample. Monofloral samples had higher protein, fiber, tocopherol, and total phenol content, along with higher ABTS and ORAC values, but lower carotenoid levels. In contrast, autumn samples had lower protein and lipid content but higher fiber and vitamin C levels. The predominant fatty acids were palmitic, linolenic, linoleic, and oleic acids, with most samples showing a higher proportion of polyunsaturated fatty acids (40.7–57.9%). Compared to other food matrices, the α-glucosidase inhibition values of Uruguayan bee pollen are similar to those found in raw citrus pomace. This is the first report on bee pollen’s ability to inhibit pancreatic lipase in relation to its in vitro anti-obesity properties. Uruguayan bee pollen shows significant potential for combating metabolic syndrome, obesity, and type 2 diabetes.

## 1. Introduction

Pollen is the male gametophyte of plants, collected by worker bees from flowers and mixed with their own secretions. These granules adhere to the bees’ hind legs and, upon returning to the hive, are dislodged as the bee passes through a “pollen trap” placed at the hive entrance [1]. Pollen is considered the primary food source for the growth of bee larvae [2,3].

The chemical composition, nutritional profile, and biological activity of bee pollen depend on the plant species from which it originates, as well as geographic and climatic conditions, the collection season, and storage and processing factors [4,5,6].

Bee pollen is a natural source of bioactive compounds and essential micro- and macronutrients, including carbohydrates, proteins, vitamins, amino acids, minerals, lipids, flavonoids, phenolic compounds, and essential oils [3,7,8]. It is also a rich source of essential amino acids (arginine, histidine, lysine, tryptophan, phenylalanine, methionine, threonine, leucine, isoleucine, and valine), macroelements (phosphorus, sodium, calcium, magnesium, and potassium), and microelements (copper, manganese, iron, zinc, and selenium) [1,9].

The potential health benefits of bee pollen are closely linked to its chemical composition, which varies according to its botanical and geographical origin. Evidence suggests that long-term consumption of phenolic compounds from pollen, such as flavonoids (kaempferol, quercetin, and isorhamnetin) and flavonoid glycosides, may reduce the incidence of certain cancers and chronic diseases [6,10,11].

Among the biological properties studied, bee pollen has been found to exhibit antioxidant and anti-inflammatory activity, attributed to its content of flavonoids, carotenoids, phenolic acids, and vitamins C and E [12]. Its antioxidant potential has been conferred by phenolic compounds, carotenoids (such as β-carotene), and vitamin C, which act as free radical scavengers. These compounds act synergistically to neutralize free radicals and protect cells from oxidative damage, which is essential for preventing various chronic diseases. Additionally, anti-inflammatory properties have been associated with flavonoids such as kaempferol and quercetin, which modulate inflammatory pathways by inhibiting cytokine production [11].

Moreover, bee pollen has been identified as having potential antilipemic and hypoglycemic effects. The antilipidemic effect is primarily linked to phytosterols, especially β-sitosterol, and unsaturated fatty acids like linolenic acid, which regulate cholesterol metabolism. The hypoglycemic activity has been reported for its capacity to inhibit α-glucosidase and α-amylase by polyphenolic compounds such as caffeic acid and flavonoids like kaempferol and quercetin [11]. Another notable property is its ability to inhibit the growth of pathogenic bacteria, promote the proliferation of beneficial probiotic bacteria, and support gut microbiota recovery, making pollen a valuable ally for digestive health [13,14,15]. These combined actions make bee pollen a promising natural ingredient for health promotion.

Previous studies have highlighted the importance of further research into the chemical composition of different types of bee pollen based on their botanical origins, both in relation to bee health and human health [5,16].

The Uruguayan landscape is characterized by extensive natural grasslands, agricultural areas, and scarce areas of native forests and wetlands with significant floral diversity. However, in studies on bee health and nutrition, where the botanical species used by bees have been studied and analyzed, we see that bees concentrate their efforts on highly abundant polliniferous species, generally coinciding with cultivated areas [17,18,19].

To date, no studies have characterized the physicochemical properties of pollen collected from Uruguayan apiaries. In this context, this study aimed to analyze the nutritional composition, bioactive compound profile, antioxidant activity, and enzyme inhibition capacity related to carbohydrate and fat digestion in Uruguayan bee pollen, considering its floral origin and seasonal variability.

## 2. Material and Methods

### 2.1. Bee Pollen Samples

Bee pollen samples produced by *Apis mellifera* were collected from six different apiaries during two seasons, with samples taken from the same hives in each season. Samples A1 to A6 corresponded to those collected in autumn, while samples S1 to S6 were collected in spring. Zone A1—Canelones Department (−34.763471, −55.913782), with marshes and natural grassland. Zone A2—Canelones Department (−34.674010, −56.033609), with natural grassland, native forest, and horticultural landscapes. Zone A3—Canelones Department (−34.671742, −56.184837), with natural grassland and fruit crops. Zone A4—Río Negro Department (−33.622894, −56.862813), with extensive agricultural crops. Zone A5—Artigas Department (−30.459218, −56.442190), an area with eucalyptus forests and native forest. Zone A6—Lavalleja Department (−34.526941, −55.320158), with native forest and natural grassland surroundings.

Pollen was collected using pollen traps at the hive entrance. These traps were made of plastic and disinfected with hypochlorite and then 95% alcohol. Pollen collection was carried out throughout the day [between 9:00 a.m. and 5:00 p.m.], placing three traps in three different hives and pooling the pollen collected from all three hives for analysis.

The samples were immediately frozen after harvesting and stored at −18 °C until drying. Drying was performed using a forced convection oven at 45 °C until the moisture content was reduced to below 8%. The dried samples were then stored in airtight glass containers at −18 °C until analysis.

### 2.2. Reagents

All reagents were of analytical grade. For in vitro bioactivity assays, reagents were purchased from Sigma-Aldrich (St. Louis, MO, USA): 6-Hydroxy-2,5,7,8-tetramethylchroman-2-carboxylic acid (Trolox), 2,2′-azino-bis (3-ethylbenzothiazoline-6-sulfonic acid) diammonium salt (ABTS), 2,2′-azobis (2-methylpropionamidine) dihydrochloride (AAPH), fluorescein (FL) disodium salt, 4-methylumbelliferyl-α-D-glucopyranoside, α-glucosidase from rat intestinal acetone powder, pancreatic lipase, 4-methylumbelliferyl oleate (4-MUO), and dimethyl sulfoxide (DMSO).

### 2.3. Floral Origin Determination

The pollen sample was separated into individual batches, grouping them by color. From each color group, a subsample was taken with a metal tip and mounted on a microscope slide, hydrated with a drop of water, and covered with a coverslip, following the procedure of Louveaux et al. (1978) [20]. Observation was carried out using an optical microscope with a 40× objective, identifying the natural morphology of the pollen without undergoing acetolysis. For pollen comparison and taxonomic determination, records from the palynotheca of the Faculty of Sciences at the University of the Universidad de la República [UdelaR] were used. To confirm that each color group represents a botanical taxon, a subsample of each color is taken and mounted under a microscope. When a color represents more than one taxon, the sample is homogenized with distilled water, and at least 500 pollen grains are counted and observed under a microscope. The relative frequency of each pollen type was calculated by counting a minimum of 500 pollen grains per slide.

### 2.4. Physicochemical and Bioactive Properties of Pollen Samples

Immediately before each analysis, the samples were ground and sieved through a 0.595 mm mesh.

#### 2.4.1. Proximate Composition

The moisture content of the pollen samples was determined gravimetrically by drying in a vacuum oven at 70 °C and 100 mmHg pressure until constant weight [21]. The protein content was calculated based on the nitrogen content determined using the Kjeldahl method [22], applying a factor of 6.25. The total lipid content was determined by solvent extraction (hexane/isopropanol mixture) according to Hara and Radin [23]. Briefly, 20 mL of the solvent mixture were added to 1 g of sample and stirred magnetically for 90 min. After centrifugation, the supernatant was collected and rinsed twice. The solvent was removed by rotary evaporation at reduced pressure. The ash content in the samples was analyzed by incineration in a muffle furnace at 550 °C according to de Arruda et al. [24] and determined gravimetrically. Total dietary fiber was determined using the enzymatic gravimetric method [25]. The total carbohydrate content was calculated by difference [26].

#### 2.4.2. Fatty Acid Profile

The extracted fat was derivatized according to the IUPAC 2.301 technique [27] to obtain the methyl esters (FAME). The derivatized sample was injected into a Shimadzu GC-2010 gas chromatograph (Shimadzu Corporation, Kyoto, Japan), which was equipped with a Supelco SP2560 column (100 m, 0.2 μm, and 0.25 mm) and a flame ionization detector. The temperature program used was as follows: initial temperature of 90 °C for 2 min, then ramped to 175 °C at 20 °C/min and held for 35 min, followed by a ramp to 240 °C at 15 °C/min and held for 25 min. The identification of fatty acids was carried out by comparison with a standard containing fatty acids with chain lengths ranging from C4 to C24 (Sigma-Aldrich, St. Louis, MO, USA). The results were expressed as g/100 g of fat.

#### 2.4.3. Vitamin C

The determination of vitamin C content in the pollen samples was carried out through titration with 2,6-dichlorophenolindophenol, according to the method established by AOAC [28].

#### 2.4.4. Analysis of Tocopherols

The tocopherol content was determined by liquid chromatography (HPLC) following the technique outlined by Andrikopoulos et al. [29]. Briefly, 30 mg of fat were dissolved in 1 mL of isopropanol. The sample (50 µL) was injected into a Shimadzu 20A chromatograph (Shimadzu Corporation, Kyoto, Japan) equipped with a Macherey-Nagel C18 column (250 × 4.6 mm, 100 µm) and a 20AXs fluorescence detector (λex = 290 nm and λem = 330 nm). Quantification was performed using a calibration curve with α-tocopherol. The results were expressed as µg α-tocopherol/g of fat.

#### 2.4.5. Total Carotenoids Determination

Total carotenoids were determined spectrophotometrically at 450 nm after extraction with acetone and a pre-extraction in petroleum ether [30]. The content was expressed as β-carotene equivalents (µg/g), calculated using Equation (1), where *A* is the measured absorbance, the volume of petroleum ether used in the extraction, $A_{1\mathrm{cm}}^{1\%}$, is the absorption coefficient of β-carotene in the solvent at 450 nm (2592 for petroleum ether), and *m* is the mass of the sample in grams.

(1) $$x\left(\frac{\mu g}{g}\right)=\frac{A\cdot y\left(\mathrm{mL}\right)\cdot 10^{4}}{A_{1\mathrm{cm}}^{1\%}\cdot m(g)}$$

#### 2.4.6. Instrumental Color

The instrumental color parameters L*a*b* of the different bee pollen samples were measured using a Konica Minolta CM-2300d colorimeter, as described by Machado De Melo et al. [31]. The Cartesian coordinates a* and b* were also expressed as polar coordinates: chroma (C*_ab_) and hue (h_ab_) [32].

#### 2.4.7. Total Phenol Content

Total phenol content (TPC) was determined as described by Fernández-Fernández et al. [33]. TPC analysis was performed by adding 10 µL of sample/standard to each well of translucent flat-bottom 96-well plates, followed by the addition of 200 µL of Na_2_CO_3_ (20% *w*/*v*) and 50 µL of Folin reagent (1/5). After 30 min of incubation at room temperature in the dark, absorbance was measured at 750 nm using a Thermo Scientific FC microplate reader. The results were expressed as mg of gallic acid equivalents (GAE)/g pollen through a gallic acid calibration curve (0.05–1.0 mg/mL).

#### 2.4.8. Antioxidant Properties

Antioxidant capacity by ABTS and ORAC-FL were determined as described by Fernández-Fernández et al. [33].

The ABTS method was performed by adding 10 µL of sample/standard to each well of translucent flat-bottom 96-well plates, followed by the addition of 190 µL of ABTS reagent (adjusted to an absorbance of 0.7 with 5 mM phosphate buffer, pH 7.4). After 10 min of incubation at room temperature in the dark, absorbance was measured at 750 nm using a Thermo Scientific FC microplate reader. The results were expressed as µmol of Trolox equivalents (TE)/g pollen through a Trolox calibration curve (0–1.5 mM).

The ORAC-FL method was performed by adding 20 µL of sample/standard to each well of black flat-bottom 96-well plates with a lid, followed by the addition of 120 µL of fluorescein solution (0.117 μM) and 60 µL of AAPH (48 mM). The plate was incubated for 80 min at 37 °C, and fluorescence was measured every minute (λexcitation = 485 nm, λemission = 520 nm) using a Varioskan Lux (Thermo Scientific, Waltham, MA, USA) microplate reader. The results were expressed as µmol of Trolox equivalents (TE)/g pollen through a Trolox calibration curve (10–80 µM).

#### 2.4.9. Inhibition of Enzymes Involved in Carbohydrate and Fat Digestion

The inhibitory capacity of α-glucosidase and pancreatic lipase was assessed by measuring the fluorescent probes 4-MUF-α-D-glucopyranoside and 4-methylumbelliferyl oleate, respectively, released by the action of these enzymes [34]. Fluorescence was measured (λexcitation = 360 nm, λemission = 460 nm) using a Varioskan Lux (Thermo Scientific) fluorometer microplate reader.

For the α-glucosidase inhibition assay, α-glucosidase solution was prepared by vortexing 50 mg of intestinal acetone powder from a rat in 1.5 mL of NaCl 0.9%, followed by centrifugation for 30 min at 10.000 g. The supernatant was used in a 1/10 dilution. Briefly, 100 µL of the sample, 100 µL of α-glucosidase solution, and 100 µL of 4-MUF-α-D-glucopyranoside were added to each well of black flat-bottom 96-well plates with lids. The control (maximum enzymatic activity) consisted of phosphate buffer (100 mM, pH 6.9), enzyme, and probe. Acarbose was used as the positive control, being the pharmaceutical of reference. Sample blanks were measured to subtract from the sample values. The plate was incubated for 30 min at 37 °C, and fluorescence was recorded every minute.

For the pancreatic lipase inhibition assay, pancreatic lipase solution was prepared by vortexing 8 mg of lipase from porcine pancreas type II in 1 mL of Tris-Cl buffer (10 mM, pH 8–8.4). Briefly, 50 µL of the sample, 50 µL of pancreatic lipase solution, and 100 µL of 4-methylumbelliferyl oleate were added to each well of black flat-bottom 96-well plates with lids. The control (maximum enzymatic activity) consisted of Tris-Cl buffer (10 mM, pH 8–8.4), enzyme, and probe. Orlistat was used as the positive control, being the pharmaceutical of reference. Sample blanks were also measured to subtract from the sample values. The plate was incubated for 30 min at 37 °C, and fluorescence was measured every minute.

In both assays, dose-response curves were constructed (% Inhibition vs. [Sample] (mg/mL)) to express the results as IC50 values (mg/mL).

### 2.5. Statistical Analysis

The proximate composition, along with the content of vitamin C, total tocopherols, carotenoids, and antioxidant capacity, was analyzed using analysis of variance (ANOVA). Sample, season, and monofloral characteristics were considered as fixed sources of variation. Tukey’s post-hoc test was used to compare means and identify significant differences (*p* ≤ 0.05) between samples across all assays. Principal component analysis (PCA) was applied as a dimensionality reduction technique to visualize the results. All statistical analyses were performed using XL Stat 2021.7 software (Addinsoft, New York, NY, USA).

## 3. Results and Discussion

### 3.1. Floral Origin

Table 1 and Table 2 present the botanical origin of the pollen samples collected in autumn and spring, respectively. Among the autumn samples, three were monofloral: A2 (monofloral *Casuarina cunninghamiana*), A5, and A6 (monofloral *Eucalyptus* sp.) (Table 1).

The spring samples were mostly multifloral, with the exception of sample S4, which was monofloral from rapeseed, a typical crop at the end of winter in Uruguay (Table 2). Pollen samples from spring had a broader floral spectrum compared to autumn, with an average of 9.5 botanical species per sample, compared to 5.3 in the autumn samples. Pollen typical of Uruguay’s landscape was present in all samples, which highlights the importance of cultivated resources in abundant pollen production. Whenever a floral type represented more than 45% of the total analyzed, it corresponded to a cultivated resource, such as casuarina, eucalyptus, and rapeseed. A total of 34 pollen taxa were recorded: 22 native, 11 exotic, and 1 unidentified. Families such as Salicaceae (willows) and Asteraceae, were particularly abundant among the native resources for their high pollen production and widespread presence in the areas and collection dates.

### 3.2. Physicochemical Analysis

According to the results presented in Table 3, all the dehydrated pollen samples had a moisture content of less than 8%, meaning that the pre-treatment of drying at 45 °C was effective in meeting the specifications established in national regulations [35]. Lowering the moisture content of freshly collected pollen is important to extend its shelf life at room temperature, as fresh pollen provides an ideal environment for the growth of microorganisms, particularly fungi and yeasts [36]. However, there are currently different criteria regarding the maximum permissible values, ranging from 4% to 8% [37,38,39]. Several countries have established quality standards for pollen (Argentina, Brazil, Poland, and Switzerland) that differ in terms of the ranges or maximum limits of certain parameters. Therefore, it would be important to standardize criteria that facilitate the commercialization of this product across different countries, as proposed by Campos et al. [40].

Protein is the second most present macronutrient after carbohydrates, with values that range from 16.77% to 27.26% (on a dry matter basis (DM)). and an average of 21.11%. These results are similar to those reported by Santos et al. [16] in a study conducted in Uruguay and those reported by Gasparotto Sattler et al. [41] in pollen samples from southern Brazil. Gardana et al. [42] also reported an average protein content of 21.6% and 19.5% for samples of Colombian and Italian origin, respectively. In our study, the protein content was significantly influenced (*p* = 0.0466) by the harvest season, with autumn samples having a significantly lower average protein content (19.24%) compared to spring samples (21.14%). This is consistent with the presence of Asteraceae species typical of the Uruguayan landscape, which in previous studies have shown to contain low protein values [16]. Other authors have reported that one of the factors with the greatest influence on the protein content of pollen is its botanical origin [43]. In our study, monofloral samples had a significantly higher protein content (*p* = 0.0183) than multifloral samples (22.36% vs. 18.88%). However, it is important to highlight that the presence of all essential amino acids in bee pollen is one of the factors contributing to its high nutritional value, and therefore, the nutritional value of multifloral samples should not be underestimated [44].

The total fiber content was significantly influenced by the collection season (*p* < 0.0001) and by being monofloral (*p* = 0.0019). The autumn samples had a higher fiber content than the spring samples (15.16% vs. 12.84%). and the monofloral samples had a higher fiber content than the multifloral samples (14.89% vs. 13.11%). All values fall within the range reported by Campos et al. [40], which is from 0.3 to 20 g/100 g of pollen.

The total lipid content averaged 8.53% (DM). slightly higher than what was reported for samples from a geographically close region [41], although similar values were reported for pollen samples from Croatia [45]. Lipid content was highly significantly influenced (*p* < 0.0001) by the collection season, as autumn samples had, on average, a significantly lower lipid content (7.72%) than spring samples (10.91%).

The sample with the highest lipid content (13.17%) was S4 *, a 100% monofloral sample from the *Brassica* sp. genus (rapeseed). These results are consistent with those reported by Mǎrgǎoan et al. [46], who found high lipid content in pollen samples predominantly derived from this genus, confirming a correlation between botanical origin and pollen lipid content. Additionally, sample S4 * also had the highest proportion of linolenic acid (C18:3), accounting for 50.7% of the total identified fatty acids (Table 4). Rapeseed is a well-known source of unsaturated fatty acids, particularly oleic (C18:1), linoleic (C18:2), and linolenic (C18:3) acids, which make these results consistent with the findings from pollen analysis. The predominant fatty acids were palmitic (C16:0), linolenic (C18:3), linoleic (C18:2), and oleic (C18:1) acids (Table 4). While variations were observed among the different samples, most showed a higher proportion of polyunsaturated fatty acids (40.7–57.9%), followed by saturated (24.6–37.9%) and, lastly, monounsaturated fatty acids (6.5–14.6%). Similar results were reported by Oroian et al. [47] in pollen samples from Romania. Sample P5, a 100% monofloral *Eucalyptus* pollen, displayed a different profile, with a predominance of saturated fatty acids (31.9%), followed by monounsaturated fatty acids (27.9%), composed entirely of oleic acid, and finally, 21.1% polyunsaturated fatty acids.

Among the lipid-based compounds, carotenoid content showed the greatest variability, with results reaching up to 690 µg/g in sample S1 (multifloral). In contrast, in samples A5 * and S4 * (both monofloral, from eucalyptus and rapeseed, respectively), carotenoid presence was nearly undetectable (Table 5). These results suggest that the botanical origin of pollen significantly influences carotenoid content and that monofloral samples tend to have lower concentrations of these compounds. In this regard, Gasparotto Sattler et al. [41] also reported a wide range of total carotenoids (from 5.3 to 1233 μg/g), attributing this variability to the botanical diversity of the analyzed samples. Additionally, Oliveira et al. [48] suggested a relationship between β-carotene content and certain botanical genera and species (*Raphanus* sp., *Mimosa caesalpineafolia*, and *Macroptilium* sp.). Carotenoids are among the key components responsible for the nutritional and functional properties of pollen, as they form an important group of natural antioxidants [49]. Since they are thermolabile compounds, it is crucial that pollen drying conditions are not too harsh to prevent degradation. According to kinetic studies by Song et al. [50], drying temperatures of 40 or 50 °C—such as the one used in this study—are appropriate, as no significant changes in carotenoid content were observed. However, at temperatures of 60 or 70 °C, carotenoid content drops drastically after just two hours of drying. The collection period did not affect the carotenoid content of the analyzed samples (*p* > 0.005).

Tocopherols are a group of fat-soluble compounds that exhibit vitamin E activity and play a crucial role in protecting lipid membranes from oxidative damage. They are naturally present in vegetable oils and are also used as natural antioxidants in food due to their high antioxidant potential [51,52]. The tocopherol content in bee pollen has been reported by various authors, with findings showing varying amounts. For example, Gasparotto Sattler et al. [41] reported α-tocopherol values ranging from 4.7 to 114 µg/g, with this isomer being the most abundant in all samples compared to the β, γ, and δ isomers. Tocopherol content ranged from 1.25 to 5.84 µg/g. Monofloral pollens showed a significantly higher tocopherol content (*p* = 0.0078) than multifloral pollens (4.23 vs. 2.99 µg/g). Once again, A5 * was the sample with the lowest tocopherol content, similar to its carotenoid content, suggesting a possible relationship between monofloral origin and the presence of these micronutrients.

The collection season had no significant impact; however, samples collected in autumn tended to have higher tocopherol content than those collected in spring, with an average content of 3.63 vs. 2.87 µg/g, respectively. Oliveira et al. [48] also reported similar findings, noting higher vitamin E content in samples collected in April compared to October and suggesting a link with the genera *Raphanus* sp., *Eucalyptus* sp., and the species *Mimosa caesalpineafolia*, as these were the most frequently detected in the pollen analysis of the April samples. In this study, pollen analysis indicated that sample A6 * consisted of 75.2% *Eucalyptus* and had the highest tocopherol content, aligning with Oliveira’s findings.

The vitamin C content ranged from 0.13 to 0.49 mg ascorbic acid/g. These results are similar to those reported by Gasparotto Sattler et al. [41] and Pereira De Melo et al. [53]. Vitamin C is recognized as a crucial micronutrient required by the human body, acting as an antioxidant and contributing to the cellular function of the immune system, among other important roles [54,55]. It has been reported that vitamin C content in pollen may decrease after the drying process [48]. Therefore, as with carotenoids, it is essential to properly design pretreatment processes to prevent the loss of these micronutrients. The season had a highly significant impact (*p* < 0.0001) on the vitamin C content of the samples, with autumn pollen showing a higher content than spring pollen (0.36 vs. 0.16 mg AA/g).

Regarding color, lightness ranged from 52.77 to 65.58. chroma from 34.20 to 57.06. and hue from 1.33 to 1.53. The lightness and hue values are similar to those reported by Bleha et al. [56] in Slovakian pollen. The color of Uruguayan bee pollen is more intense due to its higher Chroma value compared to what has been reported by other authors for pollen from regions such as Tunisia and India [44,57], but it is similar to that of Colombian pollen [58]. Monofloral pollens exhibited higher lightness (63.88 vs. 59.14, *p* <0.0001) and a higher hab value (1.49 vs. 1.41, *p* = 0.0004). which may have been influenced by the lower carotenoid content in these samples. as reported by other authors [31].

### 3.3. In Vitro Bioactive Properties

The results for total phenol content (TPC) and antioxidant capacity measured by ABTS (Table 6), which assess the electron transfer (ET) antioxidant mechanism, follow the same trend. The A2 * pollen sample (75.1% from *Casuarina cunninghamiana*) exhibited the highest TPC and antioxidant capacity values (*p* < 0.05), followed by P6 (75.2% from *Eucalyptus* spp.), P10 (monofloral from *Brassica* spp.), and P12 (multifloral) in terms of TPC values. For ABTS values, P5 (monofloral from *Eucalyptus*), P6, P10, and P12 showed high antioxidant capacity, with no significant differences among some of these samples (*p* > 0.05). The antioxidant capacity measured by ORAC-FL (Table 6), which evaluates the hydrogen atom transfer (HAT) antioxidant mechanism, revealed a different pattern. The highest antioxidant activity was observed in samples P2, P3 (multifloral), P4 (multifloral), P6, and P10 (*p* > 0.05). When it comes to antidiabetic activity (Table 6), sample P2 exhibited the strongest α-glucosidase inhibitory capacity (lowest IC50 value), followed by P5, P12, P9 (multifloral), and P10, with no significant differences among them (*p* > 0.05). These findings suggest that these samples have the highest potential for α-glucosidase inhibition. In contrast, sample P1 displayed the weakest inhibitory capacity. For pancreatic lipase inhibition activity (Table 6), samples P12, P2, and P6 had the highest inhibitory capacity (lowest IC50 value), with no significant differences between them (*p* > 0.05). In contrast, P4 and P3 had the lowest inhibitory capacity.

TPC values of Uruguayan bee pollen seem to be similar to the bee pollen samples reported by Mărgăoan et al. [59], Lawag et al. [60], and some of the samples reported by Aylanc et al. [1]. In contrast, Abdelsalam et al. [61] and Ilie et al. [15] reported lower TPC values, while Castiglioni et al. [62], Feas et al. [63], Soares de Arruda et al. [64], and Gabriele et al. [65] reported higher TPC values than those of Uruguayan bee pollen. Uruguayan bee pollen showed higher antioxidant capacity by ABTS than the samples reported by El Ghouizi et al. [11], but lower values than those reported by Castiglioni et al. [62]. The antioxidant capacity of Uruguayan bee pollen measured by the ORAC-FL method showed lower values than those reported by Castiglioni et al. [62], Gabriele et al. [65], and Soares de Arruda et al. [64]. Monofloral pollen samples had significantly higher TPC content (6.32 vs. 5.03, *p* = 0.0003), as well as higher ABTS values (88.39 vs. 71.62, *p* = 0.0001) and ORAC-FL values (159.12 vs. 132.43, *p* = 0.0074). Few authors have assessed α-glucosidase inhibition capacity. The α-glucosidase IC50 values of Uruguayan bee pollen were similar to those reported by Gonçalves et al. [66] (acarbose IC25 = 113.81 ± 1.00 µg/mL, IC25 = 1.19 ± 0.01 mg/mL), but higher than those reported by Khalifa et al. [67] and Laaroussi et al. [68] (lower α-glucosidase inhibition capacity). When compared with other food matrices, the α-glucosidase IC50 values of Uruguayan bee pollen measured by the same method were similar to those of raw citrus pomaces (3.42–10.84 mg/mL) [69] and lower (indicating higher inhibitory capacity) than tannat grape skin (11.67 ± 0.71 mg/mL) [33], strawberry tree extracts, and hawthorn extracts (7.26 ± 0.34 and 8.01 ± 0.27 mg/mL, respectively) [70].

As for in vitro antiobesity properties, to our knowledge, this is the first report on bee pollen’s pancreatic lipase inhibition capacity. When compared with other food matrices, Uruguayan bee pollen shows lower inhibition capacity than tannat grape skin extracts [71], strawberry tree extracts, and hawthorn extracts (8.14 ± 0.50 and 3.63 ± 0.37 mg/mL, respectively) [70]. The differences in the bioactive properties of bee pollens could be attributed to their distinct composition (vitamins C and E, carotenoids, and phenolic compounds), which may vary due to their geographical origin [11,72], and/or the method of extraction used to measure the bioactive properties. These methods may result in varying extraction efficiencies and/or the extraction of different compounds [2,60]. Differences in the chemical structure of the extracted compounds lead to variations in antioxidant and inhibition capacities [72], resulting in different values compared to those reported by other authors.

Overall, bee pollen A2 * exhibited the highest in vitro antioxidant capacity, α-glucosidase inhibitory activity, and pancreatic lipase inhibitory activity among the samples evaluated, possibly due to its content of vitamin C, phenolic compounds, and/or their combination with carotenoids and tocopherols. These results suggest that Uruguayan bee pollen has potential for counteracting metabolic syndrome, obesity, and type 2 diabetes, and particularly it may address important factors such as hyperglycemia and dyslipidemia [73]. However, further in vivo studies are necessary to confirm these findings and fully elucidate the physiological effects. The results of this study align with previously reported antidiabetic and antiobesity properties [2,8,11,67,74].

### 3.4. Principal Component Analysis

**Figure 1:**
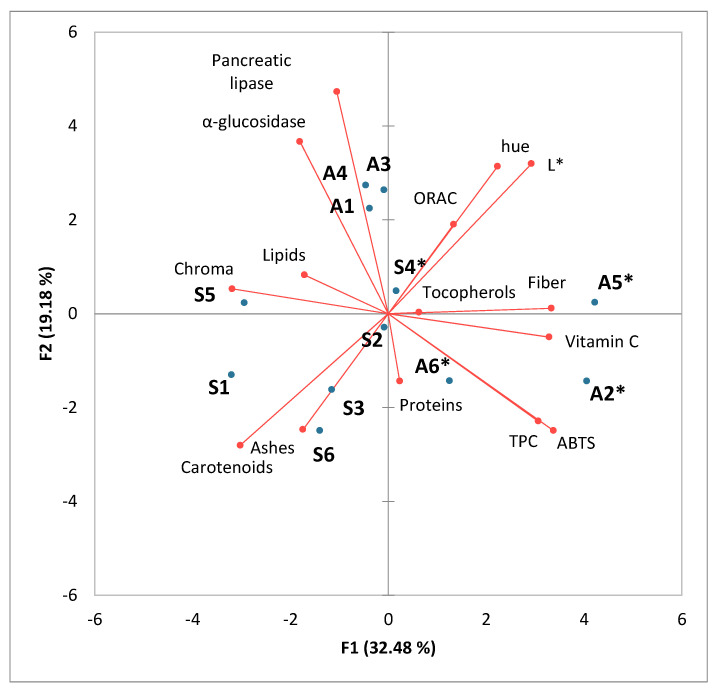
Principal component analysis (F1 and F2) carried out on physicochemical data and bioactive properties. The monofloral sample is identified with an asterisk *.

**Figure 2:**
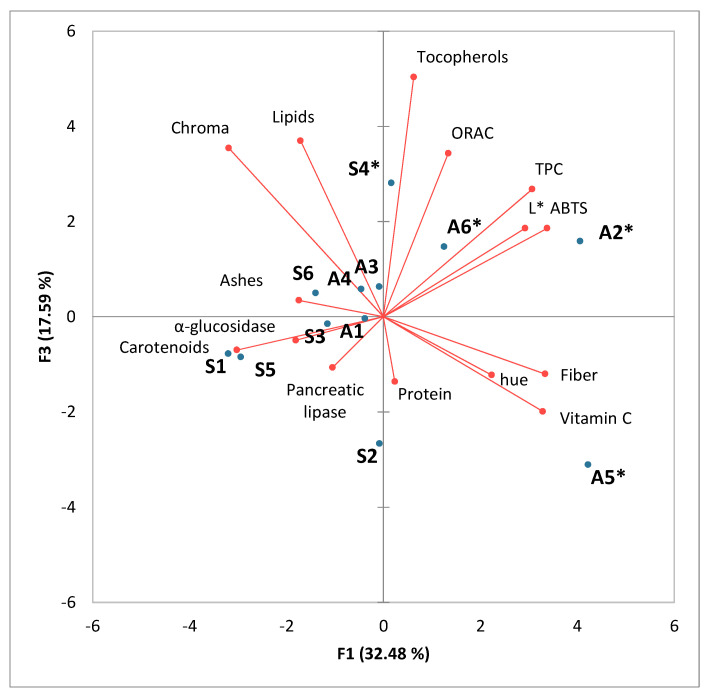
Principal component analysis (F1 and F3) carried out on physicochemical data and bioactive properties. The monofloral sample is identified with an asterisk *.

A principal component analysis was performed on the physicochemical data and bioactive properties. Moisture content was not included in the analysis as it was considered a value specific to drying rather than a characteristic of the evaluated pollens. The first three principal components accounted for 34.04%, 19.93%, and 17.59% of the variance, respectively. The PCA analysis is defined as the best linear fit for a set of variables and was specifically used in this research as a dimensionality reduction technique to explore the correlations among the variables. For a correct discussion of the results, it was necessary to consider three axes. As shown in Figure 1, the first principal component is positively correlated with total fiber content, vitamin C, luminosity, total polyphenol content, and antioxidant capacity measured by ABTS, and negatively correlated with carotenoid content and Chroma. Therefore, the samples on the right side of the first PC, such as the three monofloral samples collected in autumn, showed higher antioxidant capacity and higher content of related components like polyphenols and vitamin C, but lower carotenoid content and less color intensity. The second principal component is positively linked to hue, α-glucosidase inhibition capacity, and pancreatic lipase inhibition capacity (Figure 1), while the third principal component is positively correlated with lipid and tocopherol content, as well as antioxidant capacity measured by ORAC (Figure 2).

Figure 1 shows that the samples were quite dispersed along both PC1 and PC2, indicating that they possess different physicochemical characteristics and bioactive properties, particularly the samples collected in spring.

The positive correlation between total polyphenol content (TPC), antioxidant capacity measured by ABTS, and vitamin C content is due to the fact that both polyphenols and vitamin C are compounds with high electron-donating capacity, which contributes to the neutralization of free radicals through the electron transfer (ET) mechanism. Vitamin C, being a water-soluble antioxidant, complements the antioxidant activity of polyphenols, resulting in a synergy that increases the total activity measured by ABTS. Similar studies on the correlation between TPC and ABTS have been found in previous works with different matrices [69,71,75].

Tocopherols are lipophilic antioxidants that protect cellular membranes and lipoproteins from oxidation. They act as antioxidants by donating a hydrogen atom to peroxyl radicals, thereby stopping the lipid peroxidation chain. Since ORAC measures the efficiency of antioxidants that work under the hydrogen atom transfer (HAT) mechanism, tocopherols show a strong correlation with ORAC values [76]. The tocopherol content was also strongly associated with lipid content in component 3 (PC3). This can be explained by the fact that, as previously mentioned, being liposoluble compounds, it is expected to find a higher concentration of tocopherols in pollen samples with higher lipid content.

As expected, a positive correlation was found between Chroma and the total carotenoid content. Higher Chroma values indicate that the sample has a more vivid hue or more intense color [58], which is consistent with a higher concentration of carotenoids. The characteristic presence of a system of conjugated double bonds that absorbs light and acts as a chromophore is responsible for the yellow, orange, or red color that this type of compound imparts to foods that contain them [30]. Moreover, it was observed that the samples closest to the carotenoid variable in the graph were those collected in spring (S), which could be related to a higher production of carotenoids by plants when exposed to higher temperatures and direct solar radiation to protect themselves from photo-oxidation, as suggested by Sarungallo et al. [77].

A negative correlation can be observed between the carotenoid content and the antioxidant activity determined by ABTS. This can be explained by the positive correlation between the total polyphenol content (TPC) and the antioxidant capacity measured by ABTS (Figure 1 and Figure 2). meaning that most of the antioxidant activity is determined by this method. The positive correlation between α-glucosidase inhibition and pancreatic lipase inhibition suggests that the compounds present in the bee pollen samples may act simultaneously on both digestive enzymes. This can be explained by the presence of polyphenols and flavonoids, which have been shown to be effective inhibitors of both enzymes. Flavonoids and certain phenolic acids can interact with both enzymes through similar mechanisms, such as binding to their active sites or altering their tertiary structure, which explains the observed correlation [69].

## 4. Conclusions

Uruguayan bee pollen, based on its nutritional composition and the presence of bioactive compounds, proves to be an excellent source of proteins, polyunsaturated fatty acids, dietary fiber, vitamin C, tocopherols, and phenolic compounds with antioxidant capacity. The simultaneous inhibition of α-glucosidase and pancreatic lipase suggests a common action of polyphenols on multiple digestive enzymes. This is the first report on the pancreatic lipase inhibition capacity of bee pollen as an in vitro antiobesity property. However, the significant variation in pollen composition, driven by its different botanical origins and harvest periods, remains a challenge for promoting the bee pollen market.

In conclusion, the characterization of the nutritional composition, bioactive compound presence, antioxidant activity, and the inhibition capacity of enzymes involved in carbohydrate and fat digestion in Uruguayan bee pollen samples could be utilized to promote the production and commercialization of this apicultural product, which holds high nutraceutical properties and health benefits. Uruguayan bee pollen shows great potential in combating metabolic syndrome, obesity, and type 2 diabetes.

## Figures and Tables

**Table 1:** Botanical origin (%) of the six pollen samples collected in the fall (A_1_–A_6_). Monofloral samples are identified with an asterisk *. T—Type.

Botanical Family	Common Name	Scientific Name	A1	A2 *	A3	A4	A5 *	A6 *
Asteraceae 1	Chirca/Carqueja	*Baccharis* sp.	18.5	9.5	24.3	31.2		8.1
Asteraceae 2	Chirca/Carqueja	*Baccharis* sp.	19.5		16.1	28.4		9.3
Asteraceae 3	Chirca/Carqueja	T. *Eupatorium buniifolium*	30.3		14.7	28.8		
Myrtaceae	T. Eucalyptus	T. *Eucalyptus* spp.	8.5				100.0	75.2
Apiaceae	T. Caraguatá	T. *Eryngium* sp.	2.2		3.6			
Asteraceae	Picris	*Picris echioides*	21.0		5.2			7.1
Asteraceae	Dandelion	*Taraxacum officinale*						8.4
Arecaceae	Palm	*Butia capitata*		15.4	28.8	7.2		
Lamiaceae	Mint	T. *Mentha piperita*				2.4		
Casuarinaceae	Casuarina	*Casuarina cunninghamiana*		75.1				
Brassicaceae	Raddish	*Raphanus raphanistrum*			7.3	1.2		
Poaceae	Grass	*-*				0.8		3.2
Caprifoliaceae	Honeysuckle	*Lonicera japonica*						4.5
Fabaceae	Ibirapitá	*Peltophorum dubium*						7.3
Total General (%)			100	100	100	100	100	100

**Table 2:** Pollen origin of the six pollen samples collected in spring (%). The monofloral sample is identified with an asterisk *. T—Type.

Botanical Family	Common Name	Scientific Name	S1	S2	S3	S4 *	S5	S6
Fabaceae	Lotus	*Lotus* sp.	15.5	2.0			10.2	
Boraginaceae	Borage	*Echium plantagineum*	7.4	4.5				
Asteraceae	Senecio	*Senecio* spp.	8.1	4.8			6.7	
Myrtaceae	Eucalyptus	*Eucalyptus* sp.	15.6	25.6	28.3		12.1	15.9
Fabaceae	Red Clover	*Trifolium pratense*	1.9				4.5	
Fabaceae	White Clover	*Trifolium repens*	18.2	17.5			1.0	6.3
Caprifoliaceae	Honeysuckle	*Lonicera japonica*	5.7		2.4		4.5	3.2
Anacardiaceae	Molle	*Schinus longifolius*	14.2					6.4
Arecaceae	Palm	*-*	3	4.7			7.8	
Rosaceae	-	*-*	8.3	3.1				
Asteraceae	Chicory	*Cichorium intybus*	2.1					
Asteraceae	Carqueja	*Baccharis* sp.		10.2				
Asteraceae 1	Carqueja	*Baccharis* sp.		12.0	5.8		9.1	7.6
Asteraceae 2	Carqueja	*Baccharis* sp.		5.2				
Fabaceae	Acacia de Chaucha	*Gleditsia triacanthos*		10.4	20.2			9.8
Salicaceae	Willow	*Salix* spp.			28.7			16.7
Brassicaceae	Raddish	*Raphanus raphanistrum*			6.3			
Asteraceae	Thistle	T. *Cirsium vulgare*					3.4	2.0
Unidentified	-	-			3.5			
Fabaceae	Cina cina	*Parkinsonia aculeata*			4.8			
Apiaceae	Caraguatá	T. *Eryngium horridum*.					8.9	
Brassicaceae	Rapeseed	*Brassica* spp.				100.0		
Liliaceae	-	-					9.0	
Onagraceae	Water Flower	*Ludwigia peploides*					2.3	
Asteraceae	Picris	*Picris echioides*					12.3	
Sapindaceae	Chal-chal	*Allophylus edulis*					8.2	10.2
Cannabaceae	Tala	*Celtis* sp.						6.4
Myrtaceae	Pitanga	*Eugenia uniflora*						13.1
Fabaceae	Ñapinda	*Acacia bonariensis*						2.4
Total General			100	100	100	100	100	100

**Table 3:** Proximal composition of the 12 pollen samples analyzed *.

Sample	Moisture (%)	Protein (%)	Lipids (%)	Ash (%)	Total Fiber (%)
A1	7.69 ± 0.02 ^f^	16.87 ± 0.22 ^b^	8.49 ± 0.06 ^ef^	1.95 ± 0.02 ^ab^	14.98 ± 0.15 ^e^
A2 *	6.05 ± 0.05 ^a^	16.77 ± 0.44 ^b^	9.15 ± 0.25 ^g^	1.94 ± 0.01^ab^	16.76 ± 0.11 ^f^
A3	7.44 ± 0.19 ^ef^	16.87 ± 0.37 ^b^	8.41 ± 0.23 ^def^	2.02 ± 0.03 ^ab^	13.10 ± 0.21 ^cd^
A4	7.45 ± 0.16 ^ef^	17.43 ± 0.20 ^b^	9.01 ± 0.12 ^fg^	1.90 ± 0.02 ^a^	12.95 ± 0.12 ^bc^
A5 *	6.80 ± 0.08 ^bc^	23.26 ± 0.12 ^c^	4.37 ± 0.34 ^a^	2.06 ± 0.03 ^bc^	18.60 ± 0.16 ^g^
A6 *	6.73 ± 0.12 ^bc^	24.21 ± 0.27 ^cd^	6.90 ± 0.38 ^b^	2.18 ± 0.01^cd^	14.55 ± 0.15 ^e^
S1	7.40 ± 0.18 ^def^	24.72 ± 0.55 ^d^	8.24 ± 0.22 ^cde^	2.70 ± 0.02 ^f^	13.47 ± 0.12 ^cd^
S2	6.51 ± 0.21 ^b^	23.54 ± 0.20 ^cd^	7.69 ± 0.13 ^c^	2.40 ± 0.03^e^	13.12 ± 0.13 ^cd^
S3	7.35 ± 0.16 ^def^	24.40 ± 0.10 ^cd^	8.73 ± 0.11 ^efg^	2.93 ± 0.05 ^g^	10.18 ± 0.19 ^a^
S4 *	7.06 ± 0.20 ^cd^	23.31 ± 0.12 ^c^	13.17 ± 0.20 ^i^	2.80 ± 0.01 ^fg^	13.14 ± 0.17 ^cd^
S5	6.67 ± 0.03 ^b^	17.25 ± 0.38 ^b^	10.73 ± 0.12 ^h^	2.17 ± 0.08 ^cd^	12.46 ± 0.22 ^b^
S6	7.24 ± 0.12 ^de^	13.62 ± 0.15 ^a^	7.86 ± 0.23 ^cd^	2.20 ± 0.04 ^d^	13.52 ± 0.18 ^d^
Significance Level	<0.0001	<0.0001	<0.0001	<0.0001	<0.0001

* Samples indicated with (*) were classified as monofloral. Means with a common letter in the same column are not significantly different (*p* > 0.05) according to the Tukey test.

**Table 4:** Fatty acid profile of pollen samples.

Fatty Acid	Sample											
	A1	A2 *	A3	A4	A5 *	A6 *	S1	S2	S3	S4 *	S5	S6
4:0	0.1	-	0.2	0.3	0.1	0.1	0.1	0.2	0.2	0.1	0.1	0.1
6:0	0.3	0.1	0.1	0.1	0.1	0.1	0.3	0.3	0.2	0.1	0.2	0.2
8:0	2.0	0.2	1.0	1.3	0.2	0.5	0.2	0.9	0.3	0.1	0.8	1.2
10:0	0.4	0.1	0.2	0.8	0.8	0.5	0.8	0.8	0.5	0.2	0.4	0.2
12:0	1.2	0.2	0.3	0.7	0.6	2.8	2.0	1.3	1.0	0.4	2.4	0.5
14:0	0.6	0.3	0.6	1.3	1.8	0.9	1.7	3.3	2.4	2.5	0.3	1.0
16:0	22.6	27.9	22.1	22.8	10.4	16.2	19.3	22.5	18.8	17.9	21.3	22.5
17:0	1.3	0.2	1.0	1.1	1.3	2.4	3.8	2.6	2.2	5.8	2.7	1.1
18:0	2.0	2.5	2.9	3.5	4.2	2.0	3.4	4.5	3.1	2.1	2.6	6.8
18:1 n-9	8.6	10.6	15.5	9.9	27.9	13.0	11.2	12.1	12.1	9.1	6.1	11.3
18:2 trans	1.8	-	2.2	1.9	5.6	3.5	1.2	1.1	3.9	1.5	0.9	2.9
18:2 c n-6	19.0	39.5	17.5	19.7	15.7	16.3	15.8	14.4	11.4	5.6	14.6	24.3
20:0	0.5	0.4	0.6	0.4	-	1.0	1.0	0.5	0.5	0.4	0.9	0.7
18:3 n-3	19.2	16.2	22.0	25.4	4.8	21.3	25.2	24.4	35.7	50.7	32.3	18.8
20:2 n6	0.8	-	-	-	1.7	2.0	1.1	0.8	0.6	-	1.3	1.1
22:0	0.6	0.3	0.7	0.7	-	0.6	0.8	0.3	0.3	0.1	0.7	0.9
20:3 n-6	0.4	-	0.6	-	0.4	0.5	-	-	-	-	-	-
20:3 n-3	2.2	0.1	0.7	0.4	-	-	0.7	0.3	0.1	0.6	0.9	0.8
23:0	0.4	0.1	0.5	0.4	0.4	0.6	0.8	0.2	0.2	0.2	0.2	0.2
22:2 n6	1.6	0.1	0.8	0.8	0.7	2.4	1.2	0.1	0.3	0.1	0.3	0.4
24:0	2.9	0.2	1.2	0.7	0.1	0.3	0.6	0.8	0.2	0.1	0.7	1.2
20:5 n-3	0.3	-	0.4	0.2	0.7	0.4	0.9	0.4	0.2	1.0	0.3	0.2
24:1 n-9	1.2	0.1	0.2	0.1	0.1	0.3	0.6	0.5	0.1	0.2	0.4	0.2
22:6 n-3	0.6	0.1	0.9	1.2	2.0	1.2	0.4	0.4	0.1	0.1	0.5	0.4
Total identified	90.6	99.2	92.5	93.8	91.4	90.0	93.1	92.7	94.3	98.8	91.8	96.8
Unidentified	9.4	0.8	7.5	6.2	8.6	10.0	6.9	7.3	5.7	1.2	8.2	3.2
SFA (%)	34.9	32.3	31.6	34.2	31.9	27.9	34.9	38.1	29.8	30.0	33.4	36.5
MUFA (%)	9.8	10.8	15.8	9.9	28.0	14.6	11.8	12.6	12.2	9.3	6.5	11.5
PUFA (%)	41.9	55.9	42.2	47.4	25.9	44.1	45.2	40.7	48.2	57.9	49.9	45.5

* Samples indicated with (*) were classified as monofloral. SFA: Saturated Fatty Acids; MUFA: Monounsaturated Fatty Acids; PUFA: Polyunsaturated Fatty Acids.

**Table 5:** Instrumental color parameters and carotenoid, tocopherol, and vitamin C content in pollen samples.

Sample	L*	Chroma (C*_ab_)	Hue (h_ab_)	Total Carotenoid (µg/g)	Tocopherols (µg α-Tocopherol/g)	Vitamin C (mg AA/g)
A1	62.49 ± 0.97 ^fg^	54.46 ± 0.85 ^cde^	1.43 ± 004 ^c^	95.27 ± 6.04 ^e^	3.54 ± 0.26 ^f^	0.27 ± 0.01 ^d^
A2 *	63.58 ± 0.49 ^fgh^	45.29 ± 0.23 ^b^	1.49 ± 0.01 ^fg^	50.77 ± 3.48 ^c^	3.97 ± 0.24 ^g^	0.48 ± 0.01 ^g^
A3	63.92 ± 0.77 ^fgh^	53.29 ± 0.13 ^cde^	1.46 ± 0.05 ^de^	99.33 ± 4.85 ^e^	3.27 ± 0.28 ^e^	0.27 ± 0.01 ^d^
A4	61.48 ± 1.28 ^ef^	53.30 ± 1.04 ^cde^	1.44 ± 0.03 ^cd^	78.60 ± 4.60 ^d^	3.97 ± 0.32 ^g^	0.27 ± 0.01 ^d^
A5 *	64.09 ± 0.36 ^gh^	34.20 ± 0.50 ^a^	1.53 ± 0.04 ^h^	0.67 ± 0.01 ^a^	1.25 ± 0.11 ^a^	0.49 ± 0.01 ^g^
A6 *	58.84 ± 0.18 ^d^	50.97 ± 0.60 ^c^	1.37 ± 0.05 ^b^	334.70 ± 7.33 ^h^	5.84 ± 0.28 ^i^	0.40 ± 0.01 ^f^
S1	52.77 ± 0.77 ^a^	52.94 ± 0.80 ^cd^	1.35 ± 0.05 ^ab^	690.53 ± 15.55 ^k^	2.61 ± 0.23 ^c^	0.13 ± 0.01 ^b^
S2	59.16 ± 0.20 ^de^	43.68 ± 0.34 ^b^	1.48 ± 0.09 ^ef^	168.13 ± 5.60 ^f^	1.61 ± 0.15 ^b^	0.40 ± 0.01 ^f^
S3	57.27 ± 0.43 ^cd^	51.07 ± 0.05 ^c^	1.37 ± 0.04 ^b^	210.30 ± 5.50 ^g^	3.23 ± 0.27 ^e^	0.36 ± 0.01 ^e^
S4 *	65.58 ± 0.96 ^h^	55.96 ± 0.56 ^de^	1.51 ± 0.06 ^gh^	8.03 ± 2.03 ^b^	4.77 ± 0.38 ^h^	0.06 ± 0.01 ^a^
S5	55.55 ± 0.23 ^bc^	56.51 ± 0.31 ^de^	1.37 ± 0.05 ^b^	477.97 ± 12.09 ^j^	1.64 ± 0.17 ^b^	0.28 ± 0.01 ^d^
S6	53.46 ± 0.74 ^ab^	57.06 ± 0.64 ^e^	1.33 ± 0.04 ^a^	455.50 ± 10.67 ^i^	2.85 ± 0.205 ^d^	0.16 ± 0.01 ^c^
Significance Level	<0.0001	<0.0001	<0.0001	<0.0001	<0.0001	<0.0001

* Samples indicated with (*) were classified as monofloral. Means with a common letter in the same column are not significantly different (*p* > 0.05) according to the Tukey test.

**Table 6:** Results of total phenol content (TPC), antioxidant capacity (ABTS and ORAC-FL), and inhibition of digestive enzymes (α-glucosidase and pancreatic lipase) from Uruguayan bee pollen samples collected in autumn (A) and spring (S).

Sample	TPC (mg GAE/g)	ABTS (µmol TE/g)	ORAC-FL (µmol TE/g)	α-Glucosidase (IC_50_·mg/mL)	Pancreatic Lipase (IC_50_·mg/mL)
A1	5.02 ± 0.17 ^abc^	73.74 ± 8.90 ^bcd^	117.95 ± 12.46 ^bc^	8.38 ± 0.70 ^h^	28.34 ± 1.35 ^fg^
A2 *	8.49 ± 0.27 ^f^	106.03 ± 5.23 ^f^	154.15 ± 10.93 ^de^	3.88 ± 0.27 ^a^	15.49 ± 0.80 ^ab^
A3	5.16 ± 0.30 ^abcd^	71.61 ± 4.94 ^bc^	182.42 ± 15.67 ^e^	7.24 ± 0.54 ^g^	30.45 ± 2.45 ^g^
A4	4.85 ± 0.05 ^ab^	67.40 ± 0.44 ^ab^	170.20 ± 16.10 ^e^	5.71 ± 0.29 ^ef^	40.25 ± 3.74 ^h^
A5 *	5.70 ± 0.31 ^cde^	87.19 ± 3.75 ^e^	135.53 ± 10.29 ^cd^	4.53 ± 0.22 ^ab^	21.87 ± 1.77 ^cd^
A6 *	6.32 ± 0.45 ^e^	89.53 ± 7.02 ^e^	184.80 ± 6.33 ^e^	5.12 ± 0.55 ^bcde^	15.84 ± 1.67 ^ab^
S1	4.47 ± 0.12 ^a^	65.36 ± 2.67 ^ab^	139.79 ± 13.99 ^cd^	6.31 ± 0.60 ^fg^	22.11 ± 1.46 ^cd^
S2	4.88 ± 0.20 ^ab^	73.00 ± 2.72 ^bc^	81.64 ± 7.99 ^a^	5.54 ± 0.43 ^cdef^	23.99 ± 2.55 ^de^
S3	5.31 ± 0.24 ^bcd^	81.07 ± 2.31 ^cde^	113.40 ± 9.90 ^bc^	4.72 ± 0.43 ^abcd^	22.35 ± 2.17 ^fg^
S4 *	5.80 ± 0.34 ^de^	81.85 ± 1.41 ^cde^	160.08 ± 8.06 ^de^	4.76 ± 0.53 ^abcde^	18.60 ± 1.31 ^bc^
S5	4.44 ± 0.04 ^a^	56.15 ± 2.08 ^a^	110.60 ± 9.33 ^abc^	5.67 ± 0.48 ^ef^	26.40 ± 1.14 ^ef^
S6	6.21 ± 0.34 ^e^	86.72 ± 2.82 ^de^	100.19 ± 4.71 ^ab^	4.63 ± 0.42 ^abc^	12.32 ± 0.90 ^a^
Significance Level	<0.0001	<0.0001	<0.0001	<0.0001	<0.0001

* Samples indicated with (*) were classified as monofloral. All assays were carried out in quadruplicate. Means with a common letter in the same column are not significantly different (*p* > 0.05) according to the Tukey test.

## Data Availability

The original contributions presented in this study are included in the article. Further inquiries can be directed to the corresponding author.
